# Ethyl Butanoate, Constituent of Cassava Wastewater Volatiloma, a Potential Soil Fumigant Against *Meloidogyne Javanica*

**DOI:** 10.2478/jofnem-2022-0031

**Published:** 2022-09-14

**Authors:** Simone Ribeiro de Souza, Vicente Paulo Campos, Letícia Lopes de Paula, Marcio Pozzobon Pedroso, Willian César Terra

**Affiliations:** 1 Department of Plant Pathology, Universidade Federal de Lavras, Lavras, MG, Brazil; 2 Department of Chemistry, Universidade Federal de Lavras, Lavras, MG, Brazil

**Keywords:** *Meloidogyne*, nematicidal activity, organic residues, volatiles

## Abstract

As a result of more restrictive legislation regarding the use of pesticides, over the last two decades, the demand for new soil fumigants has increased. These compounds can come from a variety of sources, including organic residues. In this study, we identified in the cassava wastewater volatiloma a molecule with potential to be developed as a new fumigant nematicide. Cassava wastewater (20 mL) releases volatile compounds toxic to *Meloidogyne javanica* second-stage juveniles (J2), causing J2 mortality up to 89%. Through gas chromatography–mass spectrometry, eight compounds were identified in the cassava wastewater volatiloma, with alcohols being the major class. The compounds ethyl butanoate and butyric acid identified in cassava wastewater volatiloma were selected for *in vitro* nematicidal activities and substrate fumigation tests. The lethal concentration predicted LC^50–48 hr^ values (effective doses to kill 50% of *M. javanica* J2 population after 48 h of exposure) were 172.6 μg ∙ mL**^-^**^1^ and 301.2 μg ∙ mL**^-^**^1^ for butyric acid and ethyl butanoate, respectively. In a pot assay, the application of butyric acid and ethyl butanoate as a soil fumigant, at a dose of 0.5 mL ∙ kg**^-^**^1^ substrate, significantly (*P* < 0.05) decreased *M. javanica* infectivity and reproduction compared to the negative control (water). However, ethyl butanoate proved to be a more efficient soil fumigant (*P* < 0.05) than butyric acid, as its application reduced the number of galls and eggs to the level of the commercial fumigant Dazomet. This study is the first to demonstrate the potential of ethyl butanoate as a soil fumigant against *M. javanica*.

Root-knot nematodes (RKN; *Meloidogyne* spp.) are major threat to food production (Sikora et al., 2022). Among the many RKN species, *M. javanica* stands out as one of the four most important (Jones et al., 2013). Generally, the most reliable methods of RKN control are chemical, including fumigant nematicides (Wram and Zasada, 2019). However, the approval of more restrictive legislation related to the use of synthetic products has resulted in the withdrawal of some commonly used nematicides. For this reason, over the last two decades, the demand for new chemical products has increased (Desaeger et al., 2021).

Fumigant nematicides were the first synthesized products to be used to control plant-parasitic nematodes (PPNs) and they continue to be highly effective in reducing nematode populations in soil (Nnamdi et al., 2002; Taylor, 2003). However, active ingredients (a.i) for fumigant formulations are scarce and most of them have warning labels to indicate danger, which means that the compound is highly toxic by at least one route of exposure. New nematicides come from a variety of sources, such as chemical libraries, scientific literature, natural products, and patents (Desaeger et al., 2021). A rich source of molecules with potential to be used in the development of fumigant nematicides are in the volatilome of vegetable residues. From the salad rocket (*Eruca sativa* Mill) volatilome, erucin was identified; this is a volatile compound with LC_50–24 hr_ (effective doses to kill 50% of *M. javanica* J2 population) against *M. incognita* of 3.2 **m**g ^∙^ mL**^-^**^1^, which value is close to the predicted value for metam sodium, which is the a.i of several commercial nematicides (Wu et al., 2011; Aissani et al., 2015). An example of a volatile compound identified in the emission of plant residue that became a.i of a commercial product is the allyl isothiocyanate, which is a natural plant defense compound produced by Brassicaceae plants (Aissani et al., 2015).

Processing of cassava (*Manihot esculenta*) roots into flour or starch results in different waste products, which includes a yellowish liquid called cassava wastewater, referred to in Brazil as manipueira (Pinto et al., 2018). This liquid waste has a high content of cyanogenic glycosides, such as linamarin, which under enzymatic catalysis is turned into cyanide by a natural process called hydrolysis, releasing hydrogen cyanide (HCN), which is one of the most toxic volatile compounds to living organisms (Souza et al., 2014). The use of cassava wastewater in the control of RKN has been known for more than 40 yr (Ponte et al., 1979). The high concentration of cyanide in cassava wastewater is associated with its toxicity to nematodes. However, other constituents of cassava wastewater may also have nematicidal activity, especially the short-chain compounds, such as volatiles.

In this study, first, the nematicidal activity of the volatiles released by cassava wastewater was determined. Subsequently, we investigated the chemical characterization of the volatilome of cassava wastewater using solid-phase microextraction (SPME) followed by gas chromatography–mass spectrometry (GC–MS) analysis. Finally, *in vitro* and in vivo assays were performed to determine the fumigant potential of two volatile compounds from the cassava wastewater against *M. javanica*.

## Material and Methods

### Cassava wastewater source

The cassava wastewater used in this study was collected directly from a cassava flour factory located at Alpinópolis, Minas Gerais State, Brazil. The cassava used for the production of the flour was of the wild type, cultivar IAC 13. The collected waste yellowish liquid was kept in closed polyethylene terephthalate (PET) bottles, in a cold chamber at a temperature of 10**°**C, until it was used in the tests.

### *Meloidogyne javanica* culturing

A population of *M. javanica* was originally collected from lettuce (*Lactuca sativa*) roots at Ijaci town, Minas Gerais State, Brazil (21°16'37" S; 44° 91'66" W). The nematodes were multiplied in pots (2 L) containing commercial substrate (HT Hortaliças^®^) and tomato seedling (*Solanum lycopersicum* L. cv. Santa Clara) for 2 mon in a greenhouse with temperature ranging from 20°C to 28°C. Eggs were extracted as described by Hussey and Barker (1973). To obtain J2, a suspension of eggs was incubated in a hatching chamber at 28°C. The juveniles collected in the first 24 hr were discarded. This care was taken to standardize the J2 quality, since the aqueous suspension placed in the hatching chamber contained J2. For experiments, we only use J2 with a maximum of 48 hr of hatching.

### Nematicidal activity of volatiles released by cassava wastewater

The technique used to perform this test was developed by Barros et al. (2014). Inside Supelco^®^ vials, 35 g of autoclaved sand was deposited. On the sand surface, 20 mL of cassava wastewater was added. As a negative control, 20 mL of sterile water was used. Then, an empty microtube with a volume of 1.5 mL was partially buried in the sand. To concentrate the volatiles released by the organic residue, the vials were sealed and incubated at 28°C (± 2°C) in the dark for 3 d. After this period, an aqueous suspension of 1 mL, containing approximately 150 J2 of *M. javanica*, was injected, using a syringe, inside the microtube. Then the Supelco^®^ vials were sealed and incubated at 28°C (± 2°C) for 24 hr. At the end of this period, the vials were opened and the J2 transferred to wells of a 96-well polypropylene plate. Then, the technique developed by Chen and Dickson (2000), was used to determine whether the J2 were dead or alive. For this purpose, 10 mL of a 1.0-mol ∙ L**^-^**^1^ sodium hydroxide solution (NaOH) was added to the J2 suspension contents of each well. Juveniles that remained immobile for, approximately, 3 min were classified as dead.

## Characterization of volatile organic compounds (VOCs) released by cassava wastewater by SPME–GC–MS

To determine the volatile compounds released by the cassava wastewater, 10 mL of this liquid was placed inside 20-mL SPME flasks. The flasks were prepared in triplicate. The identification of volatile molecules was carried out using GC–MS. VOCs were extracted via headspace SPME, using Divinylbenzene, Carboxen, Polydimethylsiloxane (DVB/CAR/PDMS) fiber (Arthur and Pawliszyn, 1990). The temperature and extraction time were 55°C at 250 rpm for 35 min. The GC–MS QP 2010 Ultra mass spectrometer (Shimadzu, Japan) equipped with an AOC-5000 automatic liquid and gas injector (Shimadzu, Japan) and a HP-5 (5% phenyl to 95% dimethylsiloxane) column of dimensions 30 m × 0.25 mm × 0.25 μm was used for the separation and identification of VOCs. The temperatures of the injector, interface, and the detector ion source were 250°C, 240°C, and 200°C, respectively. The injector was operated in splitless mode or split 1:2 mode, according to the peak intensity in the samples. As a carrier gas, Helium 5.0 was used at 1.0 mL ∙ min**^-^**^1^. The temperature setting of the GC oven was 40°C to 130°C at 3°C ∙ min**^-^**^1^ and then up to 240°C at 10°C ∙ min^–1^. The mass spectrum of each chromatogram peak was extracted through the Automated Mass Spectral Deconvolution and Identification System (AMDIS) v. 2.63. The VOC identification was performed by comparing the mass spectra of the sample peaks with NIST library spectra by the Mass Spectral Search Program, and by comparing experimentally obtained retention indices (RI Exp.) with the retention indices in the literature (RI Lit.) (Adams, 2007; NIST, 2022). The experimental retention indices were obtained by injecting a homologous series of alkanes. The comparison among the mass spectra was performed only for peaks in which the similarity was greater than 80%.

### Lethal concentration (LC_50_) of butyric acid and ethyl butanoate against *M. javanica*

Based on the analysis by GC–MS, butyric acid and ethyl butanoate (Sigma-Aldrich, St Louis, MO) were selected for the tests to determine the lethal concentration (LC_50_). Butyric acid was chosen taking into account the largest peak area in the chromatogram. On the other hand, ethyl butanoate was selected due to the lack of studies of this compound against *M. javanica*. For butyric acid the concentrations used to determine the LC_50_ were: 0 μg mL**^-^**^1^, 150 μg mL**^-^**^1^, 165 μg mL**^-^**^1^, 180 μg mL**^-^**^1^, 210 μg mL**^-^**^1^, 280 μg mL**^-^**^1^, 330 μg mL**^-^**^1^, and 400 μg mL^–1^; for ethyl butanoate the concentrations used were: 0 μg mL**^-^**^1^, 100 μg mL**^-^**^1^, 150 μg ∙ mL**^-^**^1^, 200 μg ∙ mL**^-^**^1^, 250 μg mL**^-^**^1^, 350 μg ∙ mL**^-^**^1^, 450 μg mL**^-^**^1^, and 550 μg mL**^-^**^1^. These values were defined considering previous tests where the compounds were tested at concentrations of 100 μg mL**^-^**^1^ and 500 μg mL**^-^**^1^, data not shown. The different concentrations of the molecules were prepared using an aqueous solution of Tween 80 at 0.01 g mL**^-^**^1^ as a solubilizing agent. The assays were performed using 1,000-μL microtubes, in which 300 μL of aqueous suspension containing, approximately, 300 J2 were mixed to 300 μL of the stock solution of each molecule to achieve the desired final concentrations. Then, the microtubes were sealed and incubated at 28°C for 48 h. After this time period, the microtubes were homogenized, opened, and 100 μL of the suspension were transferred to a 96-well polypropylene plate for J2 observation. To find out if the immobile nematodes were dead, 10 μL of a 1.0-mol L**^-^**^1^ sodium hydroxide (NaOH) solution was added to the contents of each well. Juveniles that remained immobile for approximately 3 min were classified as dead. In this way, the mortality percentage was determined.

## Butyric acid and ethyl butanoate applied as soil fumigants

For this experiment, PET bottles of 2-L volume were used, in which 1 L of multiplant^®^ substrate was placed (60% pine bark, 15% vermiculite, and 25% humus; Terra do Paraíso; Holambra, SP, Brazil). The treatments consisted of the application of butyric acid or ethyl butanoate at a dose of 0.5 mL per liter of substrate, following the methodology described by Jardim et al. (2018). This dose is similar to the dose recommended for some fumigants in the market, e.g. allyl isothiocyanate, which is effective in reducing root-knot nematodes in soil at rate of 0.5 mL ∙ kg**^-^**^1^ and 2.0 mL ∙ kg**^-^**^1^ of soil (Wu et al., 2011). Each bottle received a 5–mL aqueous suspension containing 18,000 *M. javanica* eggs. As a positive control, the fumigant nematicide Basamid^®^ (i.a. Dazomet 980 g ∙ kg**^-^**^1^) was used, at a dose of 0.25 g ∙ L**^-^**^1^ of substrate. As a negative control, water was used. The bottles containing the mixtures were sealed with a lid and plastic film, homogenized, and kept at 28°C for 3 d. After this period, the bottles were opened and the substrate transferred to plastic trays, where it remained for 7 d to release the remaining volatiles. After this period, the substrate was then poured into 300-mL plastic cups. A 20-d-old Santa Clara tomato seedling, susceptible to *M. javanica*, was transplanted into each cup. After 60 d, the following parameters were evaluated: fresh root mass and number of galls and eggs in the root systems.

### Data analysis

All experiments used a completely randomized design and were done twice, at different times (January 2020 and September 2020). All the data sets were tested for normality (Shapiro–Wilk’s test) and homogeneity (Levene test). The experiment repetitions (experiments 1 and 2) were submitted to analyses of variance (ANOVA) and if there was no difference between them (*P* > 0.05), a combined analysis was performed. In the *soil fumigant assays*, the data of galls and eggs per gram of root were subjected to transformation via Log (x + 1) before statistical analysis to normalize the data. Once the assumptions were met, the *F*-test was applied through ANOVA. When the significance level in the *F*-test (*P* < 0.05) was reached, means of each treatment were compared with the Tukey’s test at 5% probability. In the *cassava wastewater volatile experiment*, the Student’s *t*-test (*P* < 0.05) was used to compare the means between the two groups. To determine the lethal concentration of ethyl butanoate and butyric acid required to kill 50% of *M. javanica* J2, the generalized linear regression logistic model was used through the drc package in the R program (Ritz et al., 2015). RStudio software (v.3.6.0) were used for statistical analysis and artwork.

## Results

### Nematicidal activity of volatiles released by cassava wastewater

**Figure 1:**
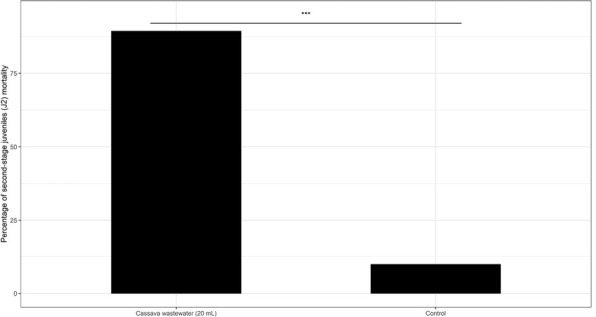
Percentage of *Meloidogyne javanica* second-stage juveniles (J2) killed after 24-hr exposure to volatiles emitted by 20 mL of cassava wastewater. The interactions between experiments and treatments were not significant (*P* = 0.063), then the data were analyzed together (*n* **=** 12). ***Significant at the 0.001 probability level according to Student’s *t*-test when compared to negative control.

Volatile compounds released by cassava wastewater were highly (*P* < 0.001) toxic to *M. javanica* J2, causing mortality above 89% (Fig. 1).

## Characterization of VOCs released by cassava wastewater by ­SPME–GC–MS

**Figure 2:**
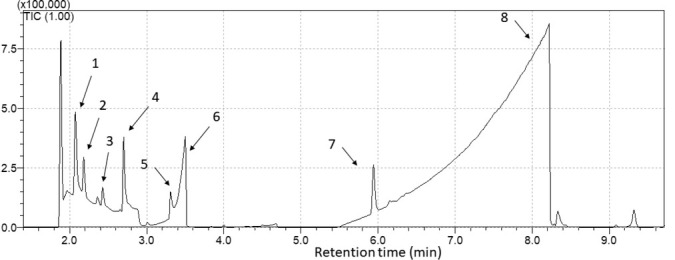
Chromatogram showing the eight compounds identified in the cassava wastewater volatiloma. **1**. ethanol, **2**. propan-2-ol, **3**. propan-1-ol, **4**. butan-2-ol, **5**. butan-1-ol, **6**. acetic acid, **7**. ethyl butanoate, **8**. butyric acid.

A total of eight compounds were identified in cassava wastewater volatiloma (Fig. 2). Most of them belong to the class of alcohols. The identified compounds are presented in Table 1, including their chemical class and peak area of each.

**Table 1:** VOCs identified in cassava wastewater by SPME–GC–MS.

Compound		Retention Time (min)	Classes	IR. Exp^a^	IR. Lit^b^	Peak area (×10^4^)
(1)	Ethanol	2.07	Alcohol	–	–	65.5
(2)	Propan-2-ol	2.19	Alcohol	–	–	23.5
(3)	Propan-1-ol	2.43	Alcohol	–	595	8.2
(4)	Butan-2-ol	2.71	Alcohol	601	598	53.1
(5)	Butan-1-ol	3.30	Alcohol	657	668	19.4
(6)	Acetic acid	3.50	Carboxylic acid	669	642	236.2
(7)	Ethyl butanoate	5.96	Ester	800	802	46.4
(8)	Butyric acid	8.91	Carboxylic acid	815	790	5,034.2

Experimental retention indices calculated by injecting a homologous series of alkanes.

Theoretical retention INDICES according to the literature.

SPME–GC–MS, solid-phase microextraction–gas chromatography–mass spectrometry; VOCs, volatile organic compounds.

### Lethal concentration (LC_50_) of butyric acid and ethyl butanoate against *M. javanica*

**Figure 3:**
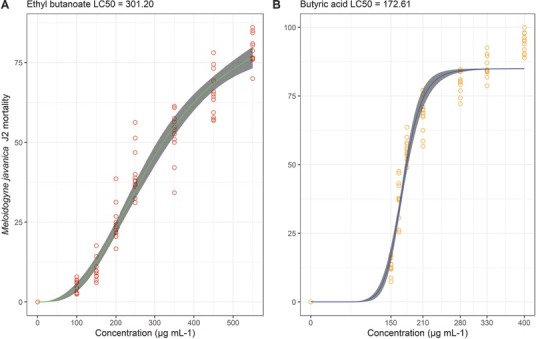
Dose-response curve of *Meloidogyne javanica* second-stage juveniles (J2) exposed to compounds with nematicidal activity. *M. javanica* J2 were exposed for 48 hr at 28**°**C to different concentrations of ethyl butanoate (A) and butyric acid (B). Dose-response curves were determined using a logistic model. Unfilled circles indicate the raw data collected in each experimental unit. The 95% confidence interval is represented by the shaded region around the curve. The interactions between experiments and treatments were not significant (*P* **>** 0.05), then the data were analyzed together.

The butyric acid and ethyl butanoate LC_50–48 hr_ against *M. javanica* J2 was modeled using a log-logistic regression model with four potential parameters (Table 2). Butyric acid caused mortality of 50% *M. javanica* J2 populations at a concentration 43% lower than ethyl butanoate (Table 3). None of the concentrations tested caused 100% J2 mortality (Fig. 3). Concentrations between 150 μg ∙ mL**^-^**^1^ and 210 μg ∙ mL**^-^**^1^ butyric acid caused a high increase in proportion of *M. javanica* J2 mortality (Fig. 3B)

**Table 2:** Dose-response curve log-logistic equation parameters for *Meloidogyne javanica* second-stage juveniles exposed to butyric acid and ethyl butanoate.

	Model parameter			
Compounds	*b* ^w^	*c* ^x^	*d* ^x^	*e* ^z^
Butyric acid	–9.74 (0.75)a *P* < 0.001	Fixed	Fixed	172.61 (1.15) *P* < 0.001
Ethyl butanoate	–2.65 (0.22) *P* < 0.001	Fixed	92 (0.064) *P* < 0.001	301.20 (7.12) *P* < 0.001

*b* is the coefficient denoting steepness of the dose-response curve.

*c* is the lower asymptotes of the dose-response curve (fixed at 0, in all models). *d* is upper asymptotes of the curvature, fixed at 0.85 (butyric acid).

*e* is the LC_50_ value.

In parentheses the standard error and the *P*-value for the parameters.

**Table 3:** **Concentrations (μg** ^∙^ **mL^–1^) that resulted in 50% of *Meloidogyne javanica* second-stage juveniles’ (J2) population mortality (LC_50_)**.

Compounds	Model predicted LC_50__–48 hr_(μg ^∙^ mL^–1^)^a^	
Butyric acid	172.61 (170.34–174.87)	
Ethyl butanoate	301.20 (257.82–344.58)	

The 95% confidence interval for each LC_50_ is enclosed in parentheses.

### Butyric acid and ethyl butanoate applied as soil fumigants

**Figure 4:**
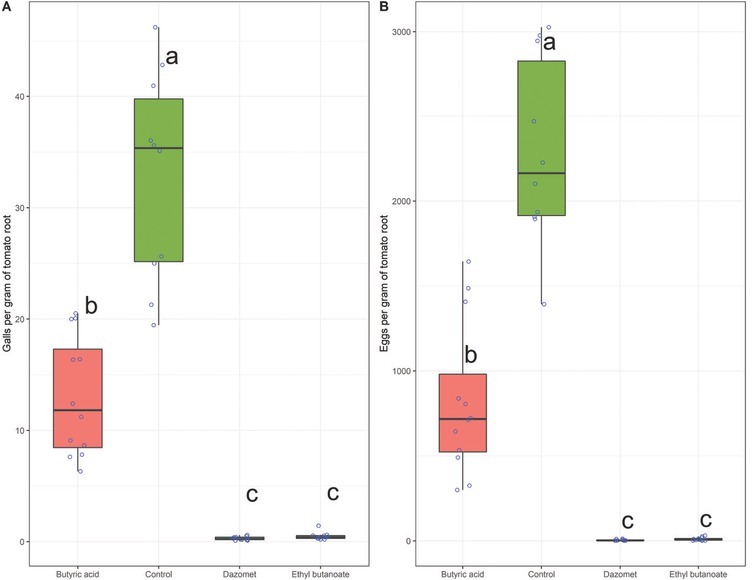
Boxplot depicting the galls (A) and eggs (B) per gram of tomato root system grown in substrate infested with *Meloidogyne javanica* in a greenhouse with temperature ranging from 20°C to 28°C. Substrate infested with *M. javanica* eggs was fumigated with butyric acid or ethyl butanoate at a dose of 0.5 mL ∙ L**^-^**^1^ of substrate. The commercial nematicide Dazomet was used as a positive control and water as a negative control. Boxes followed by the same letter do not differ by the Tukey test at 5% probability (*P* < 0.05). The interactions between experiments and treatments were not significant (*P* = 0.485), then the data were analyzed together. Unfilled circles the observations.

The application of butyric acid or ethyl butanoate, at a dose of 0.5 mL, in a substrate infested with *M. javanica*, caused a significant (*P* **<** 0.05) reduction in the number of galls and eggs per gram of tomato roots, compared to the negative control (water) (Fig. 4). However, ethyl butanoate was significantly (*P* **<** 0.05) more effective than butyric acid, the former being statistically similar to the nematicide Dazomet, used at a dose of 0.25 g ^∙^ L**^-^**^1^. The application of butyric acid or ethyl butanoate did not affect the tomato root fresh mass (RFM), being similar to the negative control (water) (Table 4). The plants grown in substrate that received the application of Dazomet presented around 37% more RFM than the plants of the other treatments.

**Table 4:** **RFM of tomato plants cultivated in substrate infested with *Meloidogyne javanica* eggs and fumigated with butyric acid or ethyl butanoate at a dose of 0.5 mL** ^∙^ **L^–1^ of substrate**.

Treatments	RFM
Ethyl butanoate	8.81 ± 1.75 b
Butyric acid	7.34 ± 1.43 b
Dazomet	11.03 ± 3.71 a
Control (water)	7.91 ± 1.85 b

Within column, means **±** standard error followed by the same letter do not differ significantly from each other by the Tukey test (*P* **<** 0.05). The interactions between experiments and treatments were not significant (*P* **>** 0.05), then the data were analyzed together. Values are means of 12 replicates per treatment. RFM, root fresh mass.

## Discussion

In the present study, using a technique that allowed exposing the nematodes exclusively to volatile compounds, it was revealed that cassava wastewater releases toxic molecules against *M. javanica* J2. Since cassava wastewater contains, among other things, HCN, nematicidal activity from this plant residue was expected (Stejskal et al., 2014). However, the literature is rich in examples showing that organic residues release a wide variety of compounds, some of them with high nematicidal activity (Aissani et al., 2015; Gonçalves et al., 2021). With this information in mind, we decided to investigate whether, in addition to HCN, cassava wastewater could emit other volatile compounds harmful to nematodes.

To confirm our suspicion, a chromatographic analysis was carried out, which identified eight compounds, most of them belonging to the alcohol class. The fact that cassava wastewater is a liquid with high organic content (up to 58.2 g ∙ L^–1^ of total sugar) and harbors several microorganisms corroborates the production of this class of chemical compounds (Damasceno et al., 2003; Vasconcellos et al., 2009). Additionally, in a previous study, organic acids, such as acetic and butyric acids, found in our chromatogram, have been identified in high concentration in cassava wastewater (Aquino et al., 2015). HCN was certainly present in the samples; however, it was not found in our analyses due to mass spectrometry limitations. The MS was operated in scan mode and only ions with mass-to-charge (m · z^–1^) between 40 a.m.u. and 350 a.m.u. were monitored in order to obtain the complete mass spectra for identification purposes. Therefore, any compound with a molecular mass lower than 40 a.m.u., such as HCN, water (H_2_O), or carbon dioxide (CO_2_), will not be detected even though it is present in the sample.

Among the compounds identified via SPME–GC– MS, ethanol and acetic acid have been extensively studied against important groups of PPNs, such as cyst nematodes and root-knot nematodes (Silva et al., 2018; Pedroso et al., 2020; Gonçalves et al., 2021). Butan-1-ol, butan-2-ol, propan-1-ol, and propane-2-ol, also present in the chromatogram, are common alcohols, with no record of toxicity to PPNs. In this work, we sought to identify the nematicidal activity of the compounds butyric acid and ethyl butanoate to support information about their potential as soil fumigants for PPNs control.

Ethyl butanoate, found in cassava wastewater volatiloma, is a short-chain ester with pineapple aroma, generally used in the food and pharmaceutical industry, with solubility in ethanol and ether. Its LC_50–48 hr_ value was calculated at 301.20 mg ∙ mL^–1^, which is considerably higher than the LC_50–24 hr_ of commercial nematicides recently placed on the market (Wram and Zasada, 2019). On the other hand, when it was applied to the infested substrate at a dose of 0.5 mL ∙ kg^–1^, practically no disease was observed. This dose is similar to the recommended dose for some commercial fumigants. For example, the allyl isothiocyanate (Allyl ITC) is effective in reducing root-knot nematodes in soil at rates of 0.5 mL ∙ kg^–1^ and 2.0 mL ∙ kg^–1^ of soil (Wu et al., 2011). Apparently, this study is the first to demonstrate, through *in vitro* and in vivo tests, the potential of ethyl butanoate as a soil fumigant against *M. javanica*.

Butyric acid was the most commonly identified compound in cassava wastewater volatiloma. This compound is a short-chain carboxylic acid, also widely used in the chemical, food, and pharmaceutical industries. The determined value of its LC_50–48 hr_ was 172.61 μg ∙ mL^–1^, which is similar to the report for the nonfumigant nematicide carbofuran, but at the same time it is considerably higher than the values reported for the new generation nematicides, e.g., fluopyram (Wram and Zasada, 2019; Fráguas et al., 2020). Previous studies have identified nematicidal activity of butyric acid on *Meloidogyne* eggs and J2. However, their effect was always inferior to that of similar fatty acids (Bansaf and Bajaf, 2003; Zhang et al., 2012). Our results corroborate those found in the literature, since the application of this compound reduced the number of *M. javanica* galls and eggs by more than 60%, when compared to the negative control.

At first we expected that the compound (butyric acid) with the best performance in the LC_50–48 hr_ tests would repeat the performance in the fumigation tests. However, the opposite occurred, since butyric acid was inferior in the soil fumigation assay compared to ethyl butanoate. The explanation for this fact is related to the physicochemical properties of these compounds, such as the vapor pressure. The higher its vapor pressure, the more volatile the liquid. As ethyl butanoate has a higher vapor pressure, it was probably better distributed in the substrate, thus finding a greater amount of *M. javanivca* eggs.

In conclusion, our results demonstrated that the cassava wastewater volatiloma is toxic to *M. javanica* J2, with alcohols being the main active volatile components of this residue. Among the two volatiles studied against *M. javanica*, ethyl butanoate stood out and showed good potential for use in the field. These studies will be done in the near future.
